# Supplementary material for: Acceptability of minimally invasive autopsy by community members and healthcare workers in Siaya and Kisumu counties, western Kenya, 2017–2018
Source: PLOS Glob Public Health. 2023 Sep 25;3(9):e0001319. doi: 10.1371/journal.pgph.0001319 (PMC10519588; doi:10.1371/journal.pgph.0001319)
Supplement: Attachment — Submitted filename: Response to Reviewers.docx [file pgph.0001319.s001.docx]

**Response to Reviewers_30 Apr 2023**

*We would like to express our appreciation for the constructive feedback provided by the reviewers regarding our manuscript. Their comments have been helpful in improving the quality of the paper. We have addressed the concerns raised by the reviewers in the revised version of the manuscript. In our response, we have highlighted some the changes made /explanations provided as follows:*

Reviewer #1: The manuscript is informative. I think please present the abstract section in structured format. please rewrite carefully the Introduction section with detail information and citied it with more references.

*While we appreciate the reviewer’s comment and suggestion, we are confused. We are unsure what “structured format” the reviewer recommends. We feel our abstract meets the PLOS Global Public Health author guidelines but would be happy to respond to this request if the reviewer could offer some more information. We have however, added some more detail and citations in the introduction section in response to the reviewer’s comments.*

Reviewer #2: GENERAL COMMENTS

A general comment is that the community members and healthcare workers appear to be in opposite on their thoughts on the procedures. This would of course be similar to other studies on the same from other contexts (case in point being the Lawrence et al study which they have also referred to); however, the presentation of the views in this context is rather not enforcing a narrative but highlighting the differences and gives an impression of two papers (one on attitudes from community members and another on attitudes from healthcare workers) in one. It would be important that they are either merged as a narrative in which it is disclosed properly either in methodology or in the reporting of the findings OR the materials from healthcare workers is actually dropped in which case if it is enough it can be used for a different paper.

*We believe that describing and discussing these differences in perspective is important within this paper rather than developing two separate manuscripts. Doing so demonstrates the diverse and differing opinions about this topic that occur within the same broader community/cultural context among different groups. Such information can inform the complexities of implementing and conducting surveillance in various contexts using the minimally invasive autopsy procedure. Therefore, we have opted to modify the manuscript to communicate this importance of describing both perspectives in the discussion of findings, specifically in the discussion on facilitators to acceptance.*

The language of the paper needs to be clear. In the introduction, it is indicated that “Here we present a qualitative assessment of the specific cultural, religious and socio-behavioural factors that influenced acceptability of post-mortem diagnostic procedures…” (lines 100 – 102), yet in reading the paper it shows that some of the participants had not had experience with the procedures and were asked in a rather hypothetical format. It would be important for the authors to highlight what really happened and what are they reporting on.

*We agree and have clarified that interviews reflected both hypothetical perspectives and perspectives that formed after the MITS procedure had been implemented.*

Also the language needs editing for coherence, for example there are references to post-mortem autopsy (e.g. line 86), an autopsy is always post-mortem. There are also references of post-mortem examination, it would be helpful just to call it autopsy or choose one word which should be used uniform across.

*In regard to the language of “post-mortem examination” we respectfully disagree with the reviewer’s suggestion and propose to retain this wording specifically because we were discussing the possibility of a procedure (a MITS) that is substantially different from the one that individuals conceptualize in their mind when asked about an autopsy. When referencing both procedures, we use the term “post-mortem examination;” when referencing only one we use the term “MITS” or “post-mortem autopsy). In addition, we propose to keep the term “post-mortem autopsy” to identify a distinct procedure from a “verbal autopsy” which is also conducted at CHAMPS sites and is referenced in the paper.*

Lines 269 – 270 “Some of the families whose children had undergone MITS were reluctant to accept results where it revealed an unfamiliar condition…” I do not think this would be reluctance to accept results other than a denial of the information and the results. Reluctance to accept results gives an impression of them not showing up for the results. But they showed up and whether they liked it or not, they got the results. In processing the results, then there can be denial or scepticism. It is a matter of language which I think across the paper needs some sort of clarity and reinforcing.

*Thank you for this comment. We asked families that had received results what they thought about those results. What we report above are cases that doubted the results and in their own views thought that the results did not reflect what, in their view, killed their child- this could influence their engagement with recommended preventive behaviour/actions. Some of these results were delivered at home. Our interpretation is that the act of showing up, on its own, did not constitute acceptance of results if the family did not agree with the results presented*. *We have however, changed the wording from “doubt” to “skeptical”*

On a general, the paper needs re-writing. There are important and interesting novel findings that it has but they are buried in a chunk of other ‘common’ findings of which some are similar to other studies from different contexts (e.g. Malawi and Mozambique). Other than focusing on the quality of the findings from the study, it feels rather that there was focus on the quantity of the findings and this is a disservice to an absolutely brilliant study which generated interesting and novel findings that has the potential of enriching the diversity of barriers as well as facilitators towards MITS and other health interventions reasons within the global health literature.

*We have added significant text to the discussion section to highlight the novel findings from our study*

PARTICIPANT SELECTION (change the subheading: I would suggest data collection)

*We have changed the subheading to Data Collection*

The first paragraph. The section is not clear. In one instance, it appears as if only Interviews (KIIs and SSI) were conducted yet again there is a mention of FGDs. Also, there is a need to highlight the actual categories of the people who were involved. They just refer to the people having some knowledge of the practices. Were they community leaders or just community members? This has been reported as a purposive sampling so there is a need to be very clear on the people who were purposively sampled.

Also, what is the difference between KIIs and SSIs? One can do a KII using a SSI, why are they treated as opposites in this reporting when the opposite of semi-structured is structured? In the lines 133-134 there are the words “KIIs included open-ended verbal questions…” and this is just a semi-structured approach (see, for example, Fylan, F., 2005. Semi-structured interviewing. A handbook of research methods for clinical and health psychology, 5(2), pp.65-78).

*We have changed “semi-structured interview” to “in-depth interview” when describing an interview type designed to elicit perceptions from a participant that reflect those of a larger group. We reference “semi-structured interview” as a type of interview approach that reflects both key informant interviews and in-depth interviews in this study. We have added language to describe the sampling strategy and inclusion criteria.*

ANALYSIS

This study has been reported as having used a grounded theory analysis. However, it has also reported that they had a conceptual framework in place (which is sensible considering that there was a semi-structured and structured approach), it is therefore confusing how a grounded theory analysis was used.

How much influence did the framework have? How different was the data from the interviews to the recorded data from observations in as far as the study objectives are concerned that they had to have two different, and even contradictory, approaches for analysis? In the way that the results are presented and subsequently discussed, there is little evidence of a grounded theory analysis other than a thematic analysis. The authors should clarify on that within the analysis section which at this point is not clear.

*We have clarified that grounded theory informed the method for the research on beliefs, activities, and rituals that accompany death and burial whereas a phenomenological approach informed the more directed research on perceptions about MITS.*

BACKGROUND CHARACTERISTICS

Had a child enrolled in CHAMPS? Why is there the NA option? You can either have a child enrolled or not. That part on the table is not clear.

*There were categories of participants who were sampled for this qualitative study based on their professions, work streams and experiences or knowledge, but were not eligible to participate in the child mortality surveillance because they were not residents of the delimited CHAMPS study catchment area/ health and demographic surveillance system. In such cases, the question of whether they had had a child enrolled in CHAMPS was not relevant and was not put to them. We believe this is different than those who were eligible but had not had a child enrolled in CHAMPS. N/A option refers to cases where a question about having/not having a child enrolled in CHAMPS was not put to the participant because the question was deemed not relevant*.

FINDINGS

KNOWLEDGE AND PERCEPTION OF POST-MORTEM

They should be separated. There is a conflation here of knowledge of autopsy and perceptions of MITS. The two are different yet that does not show in the presentation of this finding. It should be separated. Also, an engagement with the social characteristics of the respondents would provide significant and important clues. The section on the details of the participants is detailed but it does not show in the engagement with the analysis of the data. For example, as it is of qualitative research, one would expect that the categories of participant in lines 198 to 201 are identified with the aim of highlighting how his various social categories play into his response. It is not just his sex that would inform such a fatalistic position. Is he, for example, a religious leader? Do it as it has been in lines 298 – 299 where the context for the response is given. Further, the last part in lines 196 – 201, it reads like a point that belongs to the barrier aspect.

*Due to the fact that knowledge and perception can be mutually reinforcing variables in the context of autopsy, we have elected to report the two in one section. For instance, as an individual's knowledge and understanding of autopsy increases, their perception of the MITS procedure may become more positive. This in turn can lead to a greater desire to know and better understand what autopsy procedures entail. We have also made edits to this section to improve clarity.*

BARRIERS

TIMING

The heading might need to be changed just as the style of language of the paper. I take the paper as contributing to the larger body of MITS literature. The issue that is raised here, while applicable to a Kenyan experience, can however be interpreted within the broader dynamics of hospitals and politics within other settings. Broadening the conversation like that would be hugely relevant and strengthen the paper. I am thinking the barrier here would be factors beyond the MITS team, other than situating it in timing which for all practical purposes reads as if it was the timing of approach (that it is immediately after death; in which case it is discussed in the beliefs section) than that it is an issue of the political environment.

*We take note of the reviewer’s comment and we have removed the statement of the Kenyan experience and political dynamics of industrial strikes from this section*

HEALTHCARE WORKER CONCERNS

Can the concerns be split properly as those of the public and healthcare workers? The two groups have different contexts in this research. I would recommend that the first part addresses the concerns from community members and then focus is on healthcare workers concerns which appear to be little than those of community members and there is no resolve of the same in the progress of the paper.

*As we noted above, we respectfully disagree with the reviewer here. Different factors drive perceptions of the public at large and healthcare workers specifically. However, understanding perceptions of both groups is essential for the successful implementation of surveillance. We would prefer to maintain a specific description/discussion of healthcare workers’ concerns.*

CULTURAL BELIEFS

229 – 231: The sentence needs rewriting. As it is, it gives an impression that it would be acceptable in adults as it can bring them back to life.

*We have modified this sentence in response to the reviewer’s concerns.*

There needs to be an elaborate exploration of the time and periods of burial. As it is, it is rather vague and makes for difficult reading and appreciation of the context. It would be hard then to rely on such results in other contexts.

*We have made specific reference to the common cultural practice among the Luo of western Kenya, to bury young children soon after death, especially for non-hospital deaths. We have mentioned some of the beliefs that drive this practice, particularly in the case of twins.*

RUMOURS AND UNFAMILIARITY WITH AUTOPSY

These subheadings are almost communicating the same point. They are all related to the issues that have been discussed in Knowledge and perception (178 – 201). The issues discussed under the Rumours and Myths (255 – 262) can be merged with the issues on the perceptions towards MITS that are bunched together with the knowledge. The issues in the other subheading are a rehash of the arguments between 179 – 185, albeit in a different language.

The point in 269 – 276 is quite interesting that I think it should have been fully developed other than lumped together with the other repetitive point on knowledge of autopsy. It is actually a novel point from across other MITS studies and it would be relevant in the contribution of knowledge around MITS procedures.

We are report the finding about the rumour that CHAMPS program was paying bereaved families in exchange of the bodies of their children, and that such rumours mostly originated from neighbours who had not had any direct experience associated with MITS.

UNMET EXPECTATIONS

The case in 285 – 288 is similar to the case made earlier in 269 – 276, yet these fall under two different subheadings and arguments. This again points to the need of harnessing the paper. These are also the interesting findings that would add to the breadth of literature on MITS and would even be applicable to other health interventions.

*We have added significant text to towards the end of this section to help with clarity*

TIMELY PERFORMANCE

320 – 324: the quotation is not related to the argument advanced. Was the religious leader speaking as someone who had consented to MITS? The language in the sentences preceding the quote appear to indicate that this is a discussion on the reasons for consent to MITS, not what would be the probable reasons. In addition, the quotation coming after it is as well not communicating the same thing as the argument. The quotations are at best from people who did not consent for MITS yet the argument is for the reasons for consent to MITS.

*We have rephrased these sentences to clarify that an inability to carry out MITS in a timely manner would lead to refusal by complicating the timing of carrying out the ritual practices described in the quotes that take precedence for community members. Because the site has streamlined the process so as to minimize such conflict, such efforts are a facilitator for MITS acceptance*.

DISCUSSION

Point between 370 to 372 is interesting as it contradicts studies in other contexts e.g. I would recommend that the authors engage with that literature as well which they have not done despite this being a discussion section. Also, a significant focus on such findings (which are not highlighted in the findings section as elaborately) would be important.

There is also an overlap between the discussion and recommendations such that the discussion is not properly developed. I recommend that the two areas are split in which the discussion is a section on its own and recommendations are in an own section. This will leave space for clear engagement with other literature as well as situating the findings within the body of relevant literature. As it is, little literature has been engaged with in the study and it shows within the discussion. Most importantly: where is the conclusion on the paper?

*We have added language and made edits to this section for clarity. While we appreciate the reviewer’s comment, we have elected to keep this section as one and to ensure that the format meets the PLOS Global Public Health author guidelines*

*Once again, we thank the reviewers for their time and effort in evaluating our manuscript, and we look forward to hearing back from you.*

*Sincerely,*

*Peter Otieno*
