# Supplementary material for: Acceptability of minimally invasive autopsy by community members and healthcare workers in Siaya and Kisumu counties, western Kenya, 2017–2018
Source: PLOS Glob Public Health. 2023 Sep 25;3(9):e0001319. doi: 10.1371/journal.pgph.0001319 (PMC10519588; doi:10.1371/journal.pgph.0001319)
Supplement: Attachment — Submitted filename: Plos GPH-D-22-01362_R1. docx.docx [file pgph.0001319.s002.docx]

The paper aims to understand barriers and facilitators to the minimally invasive tissue sampling (MITS) for cause of death ascertainment used as part of a program whose goal is to reduce child mortality in high child mortality regions. It does this quite successfully, with a number of interesting and important findings in the work. The paper represents a solid contribution to understanding of autopsy procedures in regions where they may be needed most. Findings could potentially be generalized to research investigating mortality in other populations or using different post-mortem methods for cause of death ascertainment in the region. The methods were appropriate to address the research question and it appears that the study team reached saturation of the topic through its investigations.

Despite its strengths, there are several major gaps in the paper, which once addressed, will improve the quality of the paper, and make it more useful to the reader. To be publishable, authors should: address missing details in the introduction; provide a clearer description of methods, including who participated in the activities and why so many separate activities were undertaken; provide a clear description of what was done during analysis; describe the informed consent process; address inconsistencies in the results; describe how different groups and activities compared in the discussion; clarify or remove statements in the discussion that aren't found in the results; correct references.

**Abstract**

1. Specify the geographic scale of the study in Kenya (two Health Demographic Surveillance catchment areas) and the location (north, south, east, west, central)
2. Line 26 – Revise to say “Efforts to reduce child mortality” as opposed to “Efforts to improve child mortality indicators”, as we use indicators only to track progress on reducing mortality.

**Introduction**

3. Line 26 – Unfortunately this is not quite so straightforward. Roughly half of <5 deaths occur in the first 28 days of life, and the leading two causes of death in <5s are 1. preterm birth complications and 2. Birth asphyxia/trauma. Communicable diseases like pneumonia, diarrhea and malaria follow. Thus, the statement that <5s die “largely due to diseases that are preventable and treatable” is somewhat misleading. A small revision to the language would more accurately reflect the reality of <5 mortality.
   - <https://www.who.int/news-room/fact-sheets/detail/levels-and-trends-in-child-under-5-mortality-in-2020#:~:text=Globally%2C%20infectious%20diseases%2C%20including%20pneumonia,for%20children%20under%205%20years>.
   - <https://vizhub.healthdata.org/gbd-compare/le>
4. Lines 84-87 – The transfer of bodies to or from the morgue is mentioned several times in the results in the results (Lines 278, 329, 351). Please add a description of where, when, and how this happens in the context of the study sites. This will help the reader to properly interpret the barrier/facilitator of moving the body in the results.

**Methods**

Data Collection

5. Lines 166-170 - Unclear who participated in the KIIs, IDIs and the FGDs or how participants were selected for each. Line 166, describing KIIs, says participants had “first-hand knowledge of community history, norms and values”. Later, in results, line 200, it says that 14 KII participants were parents who had experienced death of a child <5. This doesn’t necessarily fit the description described in the Methods.
6. Line 168, describing IDIs, says participants were those who represented “entities or institutions”? Several of the categories of participants described in lines 153-155 seemed to meet the qualifications for both KII and IDI participants. If administration officials and health care providers participated in IDIs, as representing, entities or institutions, and village elders, religious leaders, TBA’s and CHVs had first-hand knowledge of community history, norms, and values, this could be stated explicitly.
7. Lines 170-172 - Unclear why both IDIs and FGDs were conducted. Was the team uncertain that they would gain the data needed if just one method was used? Did they find inconsistencies between participants in IDIs they wanted to explore FGDs? Did FGDs consist of a mix of varied participants to gain diverse opinions, or were participants of similar profiles grouped together?
8. Lines 176-181 - Unclear how many of each type of field observation were conducted and why. Was it 7 total including the 3 occasions when bodies were removed to the morgue? Or were there 10 total, 7 of which included the initial visit, consent process, and removal of body from the morgue and 3 of which included only movement of the body? Was this number chosen purposively, did you reach saturation, or did the study timeline and logistics play a role?

Analysis

9. Line 186 - Need to provide more information on what happened during data analysis. Grounded theory and an inductive approach include many different tools, methods, and ways of drawing theories from the data.
   - Grounded Theory coding often involves 3 stages – 1. initial coding (line by line, performed on 2-4 transcripts), 2. Axial Coding (identifying connections in the transcripts) and 3. Focused coding (more directed, selective, conceptual, performed on all or most transcripts). Were these stages followed? If not, explain what was done.
   - Was coding and/or analysis done by one or more study team members?
   - How were results checked for qualitative rigor (credibility, transferability, dependability, confirmability)? Were there audits, review by a second study team member, study team discussions?
10. Lines 191 – 193 - Details on the guides used for data collection belong above in the section on data collection.
11. Unclear what guides were developed and what this entailed.
    - A focus group discussion would likely have a group discussion guide rather than an in-depth interview guide.
    - Did the key informants have a separate guide than the participants of the in-depth interviews or was the same guide used? If the same guide was used, should KIIs and IDIs be classed together instead of described as separate activities above in lines 156-157?
    - What was included in the field observation guide? Were field notes kept in addition, as presumably quotes in the results section came from these?
12. Translation needs to be addressed for guide development as activities were conducted in Dholuo and Kiswahili. In addition, were Dholuo and Kiswahili audio recordings transcribed in the language first and the transcription translated to English or were audio recordings transcribed directly into English?

Ethical approvals

13. Line 195-198 - The article needs to address and describe the informed consent process that was presumably undertaken with all participants. For focus group discussions, in addition to informed consent, did participants sign or agree to an agreement of confidentiality not to spread information shared in the group?
14. Lines 383-386 in Results describes a direct benefit to certain participants from joining the study, mainly parents of deceased children. This should be described in the ethics section along with a description of potential direct harms.

**Results**

15. Table 1 – In the table header, write “N (%)” in each column head. Then you can remove the % symbol in the subsequent rows in the table, making it cleaner and easier to read.
    - Unclear, why some categories have 0 (0%) and others have dashes. Please standardize or add a footnote to explain the difference.
    - Could also include the number of participants by profile here (administrative official, village elder, religious leader, TBA, CHV, healthcare provider, parents/caregivers)
16. Throughout the results, we are missing comparisons between categories of participants, activities (KIIs, IDIs, and FGDs), geographical location and background characteristics seen in Table 1. This is a large gap in the results and obscures what could be valuable work done by the research team. It would be very useful information for the reader and future researchers. Were healthcare providers more accepting or better informed of post-mortem procedures in general or did they all raise concerns about evaluation of their competencies? Did results vary by education, geographical location, sex, religion, etc.?
17. Did the study team uncover different information in the KIIs vs IDIs vs FGDs? If not, perhaps other researchers could conserve resources by conducting one or two of the activities. To this point, there do not seem to be any quotes pulled from the IDIs and few quotes pulled from field observations.
18. It is unclear if certain themes were more salient than others. Were there any barriers or facilitators that appeared more often or seemed to matter more than others? If not, this could be described in the opening paragraphs of the barriers and facilitators sections.
19. Are the sections of the results ordered by salience? If so, that could be described. If not, it would be helpful to organize the sections under barriers and facilitators in a logical order, for example addressing timing as the first section in both barriers and facilitators, healthcare worker concern/ clinical practice as the second section, to better orient the reader.
20. Lines 223 – 227 – The fear of body organ harvesting is discussed below in the section on Rumors and Myths. This text and quotation should be moved to that section to keep the same themes together.
21. Line 246, 320 - Healthcare worker strikes are mentioned. It would be helpful to have some context on whether this was an isolated event or a common occurrence in the introduction or the discussion.
22. Line 255 – The acronym “SSI” is not previously defined. Is this an IDI? If not, please define and explain in the Methods section.
23. Lines 376 – 379 – This sentence does not seem to follow the previous text. If it is describing why this was important for the study team, it can be moved to the discussion. If it is describing a viewpoint of the healthcare workers, please revise to make that clear. If the latter is true, the section title should be changed as this sentence describes community engagement, education and sensitization rather than clinical practice.

**Discussion**

24. Lines 406- 417 - The second paragraph of the discussion seems to mix up the various barriers and facilitators in the same paragraph. Likewise, this discussion would be better placed within or before paragraphs 4 and 5 which describe reaction of bereaved families to keep similar topics together.
25. Lines 406-410 – Please expand on why continued positive perception is important after the MITS procedure. The implication is that it would affect how family members describe the process to other community members and potential MITS candidates. If this is the case, it could be stated explicitly. If not, the reader needs an explanation. In addition, please state which barriers identified in the results this would address (presumably unfamiliarity with post-mortem examination and medical diagnosis?).
26. Lines 410-412 – The statement that the “study found that the potential use of MITS results to prevent similar deaths in the future or to improve child survival seemed less convincing to bereaved families.” contradicts lines 331-341 of the results, where two separate quotes from participants highlight the importance of ascertaining cause of death to prevent future <5 deaths.
27. Lines 412-414 – please move the sentence on the study in Soweto to follow the sentence in lines 409-410 “To achieve this, a detailed interactive consenting process that simplifies details of the procedure is important” as it supports this statement rather than the statement in between about improving child survival. In addition, see comment 24, as this sentence could be linked to the fourth paragraph which discusses the rarity of post-mortem autopsies in the region.
28. Lines 414-417 - Please state that this sentence supports the findings about barriers on Timing of Champs Involvement, if that is the case.
29. Discussion is missing commentary on some of the results including cultural and religious beliefs and understanding why death occurred.
30. Line 446 – Please change “feasibility” to “acceptability” as described in the study aims.
31. Lines 450-452 – The results do not describe findings of the best time to approach a family to introduce MITS. Please add the findings or remove this from the discussion
32. Lines 458-469 – Could you describe why the majority of respondents were not community members who had participated in MITS? Why did the sample include so few of the participants in MITS?

**References**

33. References 12, 23, 26 missing journal information
34. Reference 16 missing author, date, and journal information
35. Typos in references 2 and 7
