# Supplementary material for: Acceptability of minimally invasive autopsy by community members and healthcare workers in Siaya and Kisumu counties, western Kenya, 2017–2018
Source: PLOS Glob Public Health. 2023 Sep 25;3(9):e0001319. doi: 10.1371/journal.pgph.0001319 (PMC10519588; doi:10.1371/journal.pgph.0001319)
Supplement: Attachment — Submitted filename: Response to Reviewers.doc [file pgph.0001319.s003.doc]

To the Academic Editor

PLOS Global Public Health

**RE: PGPH-D-22-01362R1_ RESPONSES TO REVIEWER 3’S COMMENTS**

Thank you for giving us the opportunity to submit a revised draft of our manuscript titled *Acceptability of Minimally Invasive Autopsy by Community Members and Healthcare Workers in Siaya and Kisumu Counties, western Kenya, 2017-2018* to PLOS Global Public Health. We appreciate the effort that you and the reviewer have dedicated to providing your valuable feedback on our manuscript. We are particularly grateful to the third reviewer for the insightful comments on the paper. We have been able to incorporate changes to reflect most of the suggestions provided to the extent that we agree with them. Where we have respectfully disagreed with the reviewer, we have provided explanations on our interpretations and what informed our decision. We have highlighted the changes within the revised manuscript. Here is a point by point response to reviewer 3’s comments and concerns:

**Abstract**

Comment 1: Specify the geographic scale of the study in Kenya (two Health Demographic Surveillance catchment areas) and the location (north, south, east, west, central)

- **We have added language to the abstract specifying that the geographical location of the study was in western Kenya, specifically in Siaya and Kisumu counties.**

Comment 2: Line 26 – Revise to say “Efforts to reduce child mortality” as opposed to “Efforts to improve child mortality indicators”, as we use indicators only to track progress on reducing mortality.

- **We have revised the sentence as suggested by the reviewer.**

**Introduction**

Comment 1: Line 26 – Unfortunately this is not quite so straightforward. Roughly half of <5 deaths occur in the first 28 days of life, and the leading two causes of death in <5s are 1. preterm birth complications and 2. Birth asphyxia/trauma. Communicable diseases like pneumonia, diarrhea and malaria follow. Thus, the statement that <5s die “largely due to diseases that are preventable and treatable” is somewhat misleading. A small revision to the language would more accurately reflect the reality of <5 mortality.

- [https://www.who.int/news-room/fact-sheets/detail/levels-and-trends-in-child-under-5-mortality-in-2020#:~:text=Globally%2C%20infectious%20diseases%2C%20including%20pneumonia,for%20children%20under%205%20years](https://www.who.int/news-room/fact-sheets/detail/levels-and-trends-in-child-under-5-mortality-in-2020#:~:text=Globally%2C%20infectious%20diseases%2C%20including%20pneumonia,for%20children%20under%205%20years).
- <https://vizhub.healthdata.org/gbd-compare/le>
- **While we appreciate the reviewer’s comment and suggestion, we have interacted with recent literature, and provided additional citation (See Ref #2) that back our earlier statement that majority of the <5 deaths are due to causes that are “preventable and treatable”. We have however, slightly revised the language for clarity by replacing “a majority of the cases” to read “a substantial proportion of the cases”.**

Comment 2: Lines 84-87 – The transfer of bodies to or from the morgue is mentioned several times in the results in the results (Lines 278, 329, 351). Please add a description of where, when, and how this happens in the context of the study sites. This will help the reader to properly interpret the barrier/facilitator of moving the body in the results.

- **We have added language of description in Line 134 stating thus “In the initial stages, the site had designated only one MITS performing centre-Jaramogi Oginga Odinga Teaching and Referral Hospital in Kisumu. This meant that MITS cases enrolled at the rural site in Siaya had to be transported by road to Kisumu for MITS performance, and then back for burial”**

**Methods**

Comment 1

Methods: Data Collection

Lines 166-170 - Unclear who participated in the KIIs, IDIs and the FGDs or how participants were selected for each.

- **We have stated in Lines 157-160 that “Participants were identified using purposive sampling technique. The categories of participants included administration officials, village elders, religious leaders, traditional birth attendants, community health volunteers, healthcare providers and parents/caregivers who had previous experience with child loss**

Comment 2: Line 166, describing KIIs, says participants had “first-hand knowledge of community history, norms and values”. Later, in results, line 200, it says that 14 KII participants were parents who had experienced death of a child <5. This doesn’t necessarily fit the description described in the Methods.

- **We have added language in Line 173… to clarify that the KII participants included participants “who had experiences in proceedings related to death and dying”**

Comment 3: Line 168, describing IDIs, says participants were those who represented “entities or institutions”? Several of the categories of participants described in lines 153-155 seemed to meet the qualifications for both KII and IDI participants. If administration officials and health care providers participated in IDIs, as representing, entities or institutions, and village elders, religious leaders, TBA’s and CHVs had first-hand knowledge of community history, norms, and values, this could be stated explicitly.

- **While we appreciate this comment, we did not interpret the views from a certain participant category as representing only a single dimension, e.g. healthcare workers as representing health facilities only. Our view is that in unstructured qualitative research, one participant category may provide a wide range of viewpoints depending on their personal experiences and opinions of the subject matter, and how they relate with the wider society. For example, an administrator or village elder may provide views about community but may also have valuable opinions about what takes place at a local health facility.**

Comment 4: Lines 170-172 - Unclear why both IDIs and FGDs were conducted. Was the team uncertain that they would gain the data needed if just one method was used? Did they find inconsistencies between participants in IDIs they wanted to explore FGDs? Did FGDs consist of a mix of varied participants to gain diverse opinions, or were participants of similar profiles grouped together?

- **We have added language in Lines 194-196 to explain that the multi-method approach was aimed at facilitating data triangulation to help validate information collected by different methods regarding the acceptability and perceptions of CHAMPS activities. Triangulation in this context was useful in gaining a more complete and nuanced understanding of the phenomenon under investigation.**

Comment 5: Lines 176-181 - Unclear how many of each type of field observation were conducted and why. Was it 7 total including the 3 occasions when bodies were removed to the morgue? Or were there 10 total, 7 of which included the initial visit, consent process, and removal of body from the morgue and 3 of which included only movement of the body? Was this number chosen purposively, did you reach saturation, or did the study timeline and logistics play a role?

- **We conducted 7 field observations. The 3 events specifically mentioned (initial approach to bereaved families, consent process and body removal for burial) are part of the 7 field observations conducted.**

**Analysis**

Comment 1: Line 186 - Need to provide more information on what happened during data analysis. Grounded theory and an inductive approach include many different tools, methods, and ways of drawing theories from the data. Grounded Theory coding often involves 3 stages – 1. initial coding (line by line, performed on 2-4 transcripts), 2. Axial Coding (identifying connections in the transcripts) and 3. Focused coding (more directed, selective, conceptual, performed on all or most transcripts). Were these stages followed? If not, explain what was done.

- **We employed mainly an open coding process where we engaged in line-by-line or segment-by-segment coding which included assigning descriptive labels to small sections of the data that captured the essence of the information. Coding being a flexible, dynamic and an iterative process, the team constantly revisited and refined the codes. Where possible one segment of data in a transcript could be coded to more than one theme**.

Comment 2: Was coding and/or analysis done by one or more study team members?

How were results checked for qualitative rigor (credibility, transferability, dependability, confirmability)? Were there audits, review by a second study team member, study team discussions?

- **The study team together developed the codebook and agreed on a description of each thematic code. Coding was done by four assistant research officers who had also taken part in conducting the interviews, and later reviewed by the study supervisor. Similarly, analysis was done by the study team with intermittent discussions to generate meaningful interpretations and findings. Such discussions ensured that there was consensus around the results.**

Comment 3: Lines 191 – 193 - Details on the guides used for data collection belong above in the section on data collection.

Unclear what guides were developed and what this entailed.

- A focus group discussion would likely have a group discussion guide rather than an in-depth interview guide.
- Did the key informants have a separate guide than the participants of the in-depth interviews or was the same guide used? If the same guide was used, should KIIs and IDIs be classed together instead of described as separate activities above in lines 156-157?
- **In lines 220-221, we have made reference to the guides developed for IDIS, KIIs, FGDs and Field Observation. The guides were guided by thematic items contained in Fig 1. We wish to clarify that different guides were developed and used for the separate activities**

Comment 4: What was included in the field observation guide? Were field notes kept in addition, as presumably quotes in the results section came from these?

- **The field observation guide included a description of the activity being observed, location time, persons involved, what CHAMPS team said to the family and how the family responded. For activities related to body removal from morgue and burial, specific observation items included: When the ritual was carried out, what time of day, how long after the death of the child, what events occurred before or after those events, and whether timing was important. A field observation report was compiled for each activity observed. Along with description of activity and spoken words, the reports noted any non-verbal cues or actions that occurred during the observed activity, such as wailings, sighs, pauses, tone of voice, or gestures etc.- to provide additional context for the analysis.**

Comment 5: Translation needs to be addressed for guide development as activities were conducted in Dholuo and Kiswahili. In addition, were Dholuo and Kiswahili audio recordings transcribed in the language first and the transcription translated to English or were audio recordings transcribed directly into English?

- **We have added language in Lines 201-202 to clarify that simultaneous transcription and translation was undertaken by two trained research assistants with multilingual proficiency (i.e. Dholuo, Kiswahili and English).**

**Results**

Comment 1: Table 1 – In the table header, write “N (%)” in each column head. Then you can remove the % symbol in the subsequent rows in the table, making it cleaner and easier to read.

- Unclear, why some categories have 0 (0%) and others have dashes. Please standardize or add a footnote to explain the difference.
- Could also include the number of participants by profile here (administrative official, village elder, religious leader, TBA, CHV, healthcare provider, parents/caregivers)
- **We have included “N (%) in each column head and removed the % symbol in table rows. We have also provided a footnote to explain the categories that have 0 (0%) and dashes. Briefly, the 0s denote “None” while the dashes refer to instances where a particular question was deemed “Not Applicable” for a specific category, and therefore, was not asked. For example, the IDI respondents were not asked whether or not they had lost a child <5 years**

Comment 2: Throughout the results, we are missing comparisons between categories of participants, activities (KIIs, IDIs, and FGDs), geographical location and background characteristics seen in Table 1. This is a large gap in the results and obscures what could be valuable work done by the research team. It would be very useful information for the reader and future researchers. Were healthcare providers more accepting or better informed of post-mortem procedures in general or did they all raise concerns about evaluation of their competencies? Did results vary by education, geographical location, sex, religion, etc.?

- **While we take note of the reviewer’s comment that comparisons between categories of participants and other geographical/background characteristics would provide a more nuanced and insightful interpretation of the results obtained, we understand that purposive sampling as used in this formative study presents challenges in controlling for extraneous variables (that may influence the outcomes). Such lack of control over extraneous variables can undermine the reliability and validity of making comparisons between different groups or conditions. In this context, our aim of using purposive sampling was not to create a representative sample of the population but we instead focused on selecting participants who possessed certain characteristics or experiences relevant to the research question. We believe that the sample may not adequately represent the diversity and heterogeneity of the population, making it challenging to make accurate and comprehensive comparisons.**

Comment 3: Did the study team uncover different information in the KIIs vs IDIs vs FGDs? If not, perhaps other researchers could conserve resources by conducting one or two of the activities. To this point, there do not seem to be any quotes pulled from the IDIs and few quotes pulled from field observations.

- **Even where no obvious differences in opinions or views emerge, we believe that data source triangulation still played a vital role by ensuring that our findings were not unduly influenced by limitations of a single data source. Iintegrating data from various sources allowed us to corroborate our findings.**

Comment 4: It is unclear if certain themes were more salient than others. Were there any barriers or facilitators that appeared more often or seemed to matter more than others? If not, this could be described in the opening paragraphs of the barriers and facilitators sections.

- **We have added text in the introductory part of this section to clarify that among the factors reported as barriers and facilitators of acceptability, no clearly dominant patterns in the data were observed.**

Comment 5: Are the sections of the results ordered by salience? If so, that could be described. If not, it would be helpful to organize the sections under barriers and facilitators in a logical order, for example addressing timing as the first section in both barriers and facilitators, healthcare worker concern/ clinical practice as the second section, to better orient the reader.

- **We prefer to maintain the order of presenting the barriers first, followed by facilitators. However, we wish to state that this should not be interpreted to mean that the issues around barriers were weightier than those around facilitators. We recognize that research is a cumulative process, and even non-salient findings can provide valuable insights and contribute to the advancement of knowledge about MITS implementation and its acceptability. We believe that by approaching non-salient findings with honesty, critical thinking, and a focus on learning, our findings still make meaningful contributions to understanding contextual issues around MITS acceptability.**

Comment 6: Lines 223 – 227 – The fear of body organ harvesting is discussed below in the section on Rumors and Myths. This text and quotation should be moved to that section to keep the same themes together.

- **We agree with this comment and we have moved the quotation to the section on “rumours and myths”**

Comment 7: Line 246, 320 - Healthcare worker strikes are mentioned. It would be helpful to have some context on whether this was an isolated event or a common occurrence in the introduction or the discussion.

- **Whereas the need for context is important, we are careful not to distract the reader by introducing more detail or language about a different focus that may detract from the factual presentation of our findings The illustrative quote from the respondent refers to her experience during a strike. This may have been a one-time experience but if an experience evokes strong emotions like fear, mistrust, or sadness, it can leave a lasting impression on an individual's mind. Such experiences can spur emotions that can lead to deeply ingrained beliefs. We have, however, revised our statement to refer to an “an industrial strike by healthcare workers” that took place during this study period.**

Comment 8: Line 255 – The acronym “SSI” is not previously defined. Is this an IDI? If not, please define and explain in the Methods section.

- **We have deleted the word SSI and replaced it with IDI**

Comment 9: Lines 376 – 379 – This sentence does not seem to follow the previous text. If it is describing why this was important for the study team, it can be moved to the discussion. If it is describing a viewpoint of the healthcare workers, please revise to make that clear. If the latter is true, the section title should be changed as this sentence describes community engagement, education and sensitization rather than clinical practice.

- **We have moved this sentence to the discussion section. However, we wish to clarify that this sentence explains the importance of engaging with healthcare workers as part of this formative research.**

**Discussion**

Comment 1: Lines 406- 417 - The second paragraph of the discussion seems to mix up the various barriers and facilitators in the same paragraph. Likewise, this discussion would be better placed within or before paragraphs 4 and 5 which describe reaction of bereaved families to keep similar topics together.

- **The paragraph highlights the importance of a simplified MITS consenting process. In the same paragraph, we discuss how this finding compares with previous literature. We have clearly identified similarities between our results and those reported in the literature. We would prefer to maintain this flow and coherence in this section.**

Comment 2: Lines 406-410 – Please expand on why continued positive perception is important after the MITS procedure. The implication is that it would affect how family members describe the process to other community members and potential MITS candidates. If this is the case, it could be stated explicitly. If not, the reader needs an explanation. In addition, please state which barriers identified in the results this would address (presumably unfamiliarity with post-mortem examination and medical diagnosis?).

- **We agree with the reviewer and have added language in the paragraph to explain need for continued positive perception.**

Comment 3: Lines 410-412 – The statement that the “study found that the potential use of MITS results to prevent similar deaths in the future or to improve child survival seemed less convincing to bereaved families.” contradicts lines 331-341 of the results, where two separate quotes from participants highlight the importance of ascertaining cause of death to prevent future <5 deaths.

- **We have added significant text to the paragraph that acknowledges the inconsistency and our interpretation that such inconsistency may represent or result from unfamiliarity with the science of post mortem and its value in child mortality surveillance, or just a case of divergent opinions by different community members on a single issue.**

Comment 4: Lines 412-414 – please move the sentence on the study in Soweto to follow the sentence in lines 409-410 “To achieve this, a detailed interactive consenting process that simplifies details of the procedure is important” as it supports this statement rather than the statement in between about improving child survival. In addition, see comment 24, as this sentence could be linked to the fourth paragraph which discusses the rarity of post-mortem autopsies in the region.

- **We have moved the sentence and positioned it as suggested by the reviewer**

Comment 5: Discussion is missing commentary on some of the results including cultural and religious beliefs and understanding why death occurred.

- **We have inserted text in the discussion about cultural and religious beliefs**

Comment 6: Line 446 – Please change “feasibility” to “acceptability” as described in the study aims.

- **We have changed the word as suggested by the reviewer**

Comment 7: Lines 450-452 – The results do not describe findings of the best time to approach a family to introduce MITS. Please add the findings or remove this from the discussion

- **We have inserted a statement about best timing to approach families for MITS**

Comment 8: Lines 458-469 – Could you describe why the majority of respondents were not community members who had participated in MITS? Why did the sample include so few of the participants in MITS?

- **Being a formative study, there was more interest in general community views that offered broader perspectives, beliefs or behaviours within the community. Further, there were not that many number of MITS cases already conducted to sample from. However, we acknowledge in the limitations that “views expressed in favour of or against MITS were largely hypothetical and may not mirror the actual views and perceptions that may only become clear in the face of an actual MITS situation”.**

**Ethical Approvals**

Comment 1: Line 195-198 - The article needs to address and describe the informed consent process that was presumably undertaken with all participants. For focus group discussions, in addition to informed consent, did participants sign or agree to an agreement of confidentiality not to spread information shared in the group?

- **We have added significant text to describe the consenting process**

Comment 2: Lines 383-386 in Results describes a direct benefit to certain participants from joining the study, mainly parents of deceased children. This should be described in the ethics section along with a description of potential direct harms.

- **The mentioned section refers to monetary or material contributions provided as compensation to bereaved families participating in MITS, and how the families perceived these provisions. From an ethical point of view and according to our interpretation of the local socio-cultural dynamics that obtain during bereavement, we are of the view that such contributions did not constitute “direct benefits” to participating individuals or families. Rather, we saw them as compensation as they were provided mainly as a way to mitigate any burdens or inconvenience that participants experienced as a result of their involvement in the CHAMPS program.**

**References**

Comment 1: References 12, 23, 26 missing journal information

- **We have inserted the journal information**

Comment 2: Reference 16 missing author, date, and journal information

- **We have included the missing information**

Comment 3: Typos in references 2 and 7

- **We have corrected the typos**

We look forward to hearing from you in due time regarding our submission and to respond to any further questions and clarifications that you may have.

Sincerely,

Peter Otieno

31 July 2023
